# Secondary Sex Ratio in the Face of Global Challenges: Beyond the Headlines

**DOI:** 10.3390/ijerph22111621

**Published:** 2025-10-24

**Authors:** Evangelos Axarloglou, Efthymia Delilampou, Paschalis Theotokis, Konstantinos Efthymiadis, Sofia Gargani, Maria Eleni Manthou, Soultana Meditskou, Dimosthenis Miliaras, Iasonas Dermitzakis

**Affiliations:** Department of Histology-Embryology, School of Medicine, Aristotle University of Thessaloniki, 54124 Thessaloniki, Greece; evanaxar@auth.gr (E.A.); edelilamp@auth.gr (E.D.); ptheotokis@auth.gr (P.T.); efthymiadis@gmail.com (K.E.); sgargan@bio.auth.gr (S.G.); mmanthou@auth.gr (M.E.M.); sefthym@auth.gr (S.M.); miliaras@auth.gr (D.M.)

**Keywords:** secondary sex ratio, wars, terrorism, climate change, political affairs, reproductive health

## Abstract

The secondary sex ratio (SSR), defined as the ratio of male to female live births in a population, is a crucial indicator of reproductive and public health. External factors, such as lifestyle, natural disasters, environmental chemicals and infections, have been examined as potential trendsetters of the SSR. Several global challenges have emerged in recent years, such as climate change, wars, terrorist attacks and stressful political events. These aspects can potentially impact reproductive health outcomes, fertility rates, and the overall well-being of individuals. With respect to this, they may also affect the SSR. Through an in-depth examination of the existing literature, this manuscript elucidates the complex interconnections between global challenges and the SSR. Indeed, terrorist attacks and stressful political events have been linked to a decrease in the SSR. In contrast, high temperatures and warfare have shown a propensity to elevate the SSR in numerous scenarios. However, these associations require further validation through additional studies. The precise mechanisms through which these determinants exert their influence need to be elucidated. Understanding the unseen influences of global challenges on the SSR is crucial for understanding population trends and ensuring effective public health interventions.

## 1. Introduction

**Figure 1:**
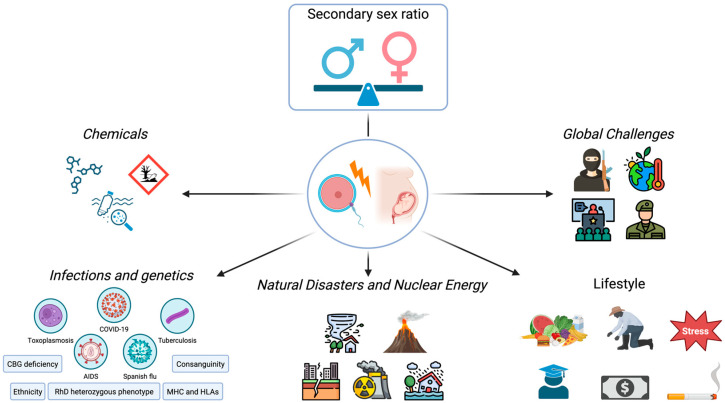
Definition and determinants of the secondary sex ratio (SSR). The SSR refers to the proportion of male to female live births. This ratio is shaped by a complex interplay of biological, environmental, and social factors. As illustrated, determinants of the SSR include chemical exposures, infections, genetic factors, natural disasters, nuclear energy, global challenges, and lifestyle factors. All influences can act either before conception or during pregnancy, affecting gamete integrity, fertilization, or fetal survival. Together, these trendsetters can shift the delicate balance between male and female birth proportions, reflecting underlying biological and environmental stressors within human populations [3,4,5,6]. Created in https://BioRender.com.

The secondary sex ratio (SSR) refers to the proportion of male to female live births and constitutes a critical demographic parameter, often employed as an indicator of population health and biological equilibrium [1]. Globally, the SSR averages approximately 1.05, reflecting a slight predominance of male over female newborns. Deviations from the expected global mean value have long been interpreted as signals of underlying ecological, social, or health perturbations. Indeed, substantial regional and temporal variations in SSR have been observed and driven by multifactorial influences [2]. Emerging evidence, including findings from our work, suggests that factors such as parental lifestyle, exposure to environmental toxins, infectious diseases, and underlying genetic predispositions may modulate SSR (Figure 1) [3,4].

Distinct critical periods have been recognized, reflecting the substantial influence of stress on cellular and tissue differentiation [7,8,9,10,11]. Two principal windows of influence have been proposed: the moment of conception and the gestational period [12]. Hormonal milieus, particularly levels of estrogens, androgens, progesterone, and gonadotropins during the peri-conceptional phase, may bias zygotic sex determination. Specifically, elevated estrogen and testosterone levels have been associated with an increased likelihood of male offspring, while higher concentrations of progesterone and gonadotrophins appear to favour female births [13]. Furthermore, stress-induced elevations in maternal adrenal androgens are associated with male-biased fetal loss, shaping the SSR at birth [14,15]. From an evolutionary perspective, the Trivers–Willard hypothesis posits that parental condition influences offspring sex allocation, favouring males when parental fitness is high [16,17]. Notably, the precise mechanisms underlying these effects remain a subject of ongoing scientific inquiry [18].

In recent years, humanity has encountered an unprecedented convergence of multiple large-scale stressors, each exerting significant and multidimensional effects on societal structures and population dynamics [19]. Armed conflicts and military interventions function as major destabilizing factors, contributing not only to elevated mortality rates but also to economic disruption, forced displacement, and the erosion of institutional governance [20]. These conditions are associated with long-term shifts in migration patterns, infrastructure degradation, and altered demographic trajectories. In parallel, the rise of terrorism introduces persistent volatility, inducing chronic insecurity and sociopolitical fragmentation, with measurable consequences on societal stability and intergenerational behavior [21]. Political instability—characterized by governance deficiencies, ideological polarization, and geopolitical realignments—further compounds these effects by reducing institutional capacity and limiting access to essential services, thereby exacerbating social inequalities and impairing system functionality [22]. Concurrently, climate change represents an escalating environmental stressor, manifesting through temperature increases, extreme weather phenomena, and ecological degradation [23]. These processes are linked to declining resource availability, reduced food security, and loss of habitable land, particularly in ecologically vulnerable regions. The cumulative and interactive impact of conflict, political instability, and environmental change is reshaping global demographic and societal patterns [24,25]. A systematic understanding of these interdependencies is essential for the development of effective, evidence-based adaptive strategies.

From a demographic theory perspective, fluctuations in the SSR may reflect adaptive reproductive responses to environmental and social conditions. According to this view, variations in parental condition, resource availability, and broader global stability can influence offspring sex allocation, favoring the production of one sex over the other under specific selective pressures. Although the multifaceted consequences of global challenges ranging from armed conflict to environmental degradation have been extensively examined across social, economic, and health domains, their potential influence on the SSR remains comparatively underexplored. In an era marked by persistent warfare, terrorism, political instability, and climate change, it becomes increasingly important to consider how such challenges may subtly but significantly shape reproductive outcomes.

This review endeavours to bridge that gap by critically evaluating the existing body of evidence regarding the influence of these global phenomena on SSR. By synthesizing current findings, we seek to enhance our understanding of demographic shifts under duress and to prompt further interdisciplinary research. Appreciating the nuanced interconnections between global instability and SSR variations is essential for comprehending the full scope of these challenges. Ultimately, this inquiry aims to illuminate an often-overlooked aspect of population health, offering insights into the resilience and vulnerability of human reproduction in times of profound societal change.

## 2. Review Methodology

The present review aims to comprehensively examine the influence of major global challenges, including warfare, terrorism, climate change, and political instability, on the SSR. A narrative review approach was adopted because the existing literature on global challenges and the SSR is highly heterogeneous, encompassing diverse study designs, populations, temporal frameworks, and outcome measures. This variability precludes the strict inclusion criteria and quantitative synthesis required for a systematic review. Instead, a narrative methodology allows for a comprehensive, critical, and integrative evaluation of epidemiological, biological, and socio-environmental evidence, facilitating the exploration of complex interrelations and emerging hypotheses across multidisciplinary domains. While not conducted as a systematic review, specific methodological steps were followed to ensure a rigorous and structured approach.

A literature search was performed across major academic databases, including PubMed, Scopus, and Web of Science, using combinations of keywords such as “secondary sex ratio,” “sex ratio at birth,” “wars,” “terrorism,” “climate change,” “political instability,” “stress,” and “temperature”. Relevant articles published from the inception of each database up to July 29, 2025, were considered. Reference lists of included studies were also manually reviewed to identify additional sources of relevance. Inclusion criteria encompassed peer-reviewed original articles published in English with full-text availability, focusing on factors related to global stressors and their potential effects on SSR. Studies were screened based on titles and abstracts to assess eligibility, followed by full-text evaluation. Data extracted from eligible studies included study design, population characteristics, type of global challenge analyzed, and direction or magnitude of observed SSR changes. A narrative synthesis approach was applied to integrate and interpret the findings, highlighting patterns, discrepancies, and emerging hypotheses regarding the interplay between global crises and SSR fluctuations.

## 3. The Impact of Wars on SSR

Throughout human history, the profound impact of warfare on societies has been extensive and intricate [26]. Its consequences are devastating, involving both direct and indirect casualties. Direct repercussions include loss of life, injuries, and widespread destruction of property. Indirect effects, such as psychological trauma and disruption of social cohesion, further compound the ramifications [27,28]. Notable conflicts like World War I, World War II, the Korean War and the Vietnam War are poignant reminders of warfare’s enduring impact and complexities [29,30,31]. It is essential to acknowledge that warfare also influences human reproduction [32]. However, the relationship between warfare and human reproduction may exhibit variability, underscoring the complicated nature of this association.

After World War I, several European countries observed a slight yet significant increase in the SSR during and shortly after periods of warfare [33]. Notably, Germany experienced a statistically significant increase in SSR during World War I, which later returned to the mean value. In contrast, countries following a neutral policy during World War I, like the Netherlands, presented smaller or absent SSR fluctuations. This phenomenon has been attributed to increased coital frequency, as the timing of insemination can influence the SSR [34]. The theory suggests that higher coital frequency increases the possibility of insemination in the early or late phase of the fertile period, in which gonadotropin levels are lowered, resulting in more male offspring [35]. A similar pattern of sudden SSR increase followed by a remit has been observed post-World War II. A retrospective study examining SSR alterations in the United States of America (USA) from 1922 to 1949 revealed a statistically significant upward shift in the 1942–1946 period [36]. The SSR value in 1946 was particularly elevated for first-order births, although the effect of birth order on SSR was not statistically significant. Unfortunately, the observational nature of this study, conducted in 1954, prevented the authors from identifying any underlying pathophysiological mechanisms for these outcomes.

More recently, Graffelman and Hoekstra conducted a retrospective study involving ten countries, nine European and the USA [37]. They collected yearly records starting from the beginning of the 20th century. The study assumed a potentially positive outcome of SSR in post-war periods, specifically during World War I and II. The above assumption was statistically validated using regression and randomisation methods in all cases except for Italy and Spain. Additionally, a time-series method showed a statistically significant correlation between war and increased SSR in six out of the ten countries. The authors also acknowledged the contribution of other factors correlated with the noticeable increase in SSR, such as parental age. During warfare, the age of couples who reproduce might be affected due to a higher number of casualties. However, the present study did not include co-factors to verify this hypothesis.

Another retrospective study conducted in the US examined the fluctuation of SSR in offspring born from 1940 to 1980 [38]. The study aimed to determine any probable effects of the wars that occurred during that time, namely World War II, the Korean War, and the Vietnam War. They found a statistically significant but moderate increase in SSR during wartime, with the most significant impact observed during the Vietnam War. However, the authors did not provide any physiological explanation for their findings. A different perspective on the underlying mechanism for SSR increases during wartime was presented by Claudio Bisioli [39]. He proposed an adaptive mechanism of natural selection, suggesting that the higher mortality rate of adult males during warfare, compared to females, creates an advantage for subsequent male offspring. This advantage arises due to the scarcity of males in the overall population, thus driving parental conditions to favour the birth of male offspring. However, the study did not specify the specific parental conditions, whether maternal or paternal, that are modified to support this pathway. Nevertheless, the correlation between warfare and SSR is not always positive. James WH proposes the paradigm of the ten-day war in Slovenia as contradictory evidence [40]. The observed decline in SSR during that case, it is again associated with high-stress levels [41]. Except for that, an observational study recording SSR during and after the civil war in Lebanon did not display a significant alteration in SSR [42].

**Figure 2:**
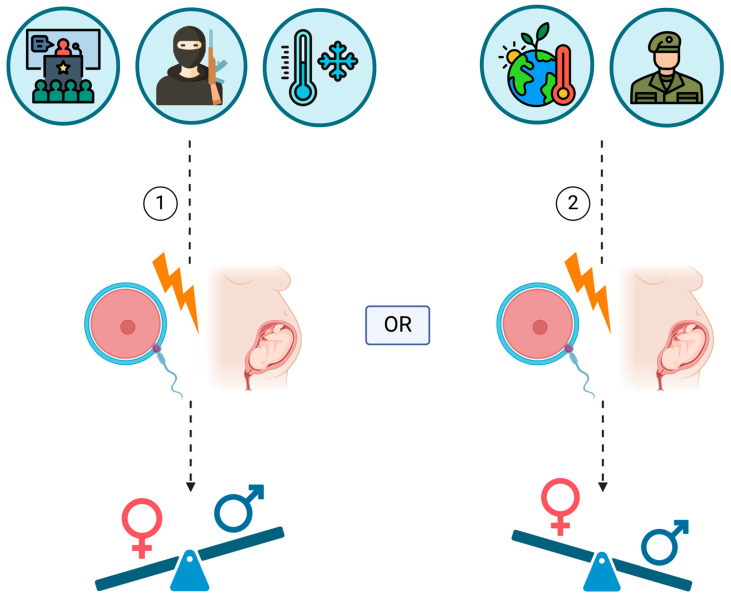
The impact of the analyzed global challenges on the secondary sex ratio (SSR). Terrorist attacks, low temperatures, and stressful political events have been linked to a decrease in the SSR (1). In contrast, high temperatures and warfare have shown a propensity to elevate the SSR in numerous scenarios (2). However, these associations warrant further validation through additional studies, especially to investigate and clarify the underlying biological mechanisms. Created in https://BioRender.com.

In conclusion, wars could have an impact on the sex ratio of offspring during and shortly after the period of warfare (Figure 2). Significant and long-term conflicts like World War I and II have shown temporary yet statistically significant increases in SSR. However, few studies have suggested a negative or any effect of wars on SSR. The upward shift in SSR could be attributed to increased coital frequency or natural selection. Nonetheless, the exact physiological mechanism behind the effect of wartime on SSR still requires further elucidation.

## 4. The Effects of Terrorism on SSR

Terrorist attacks are violent acts committed by individuals or groups and can be expressed through a variety of means, such as bombing and mass shooting. Thus, they are considered external stressors and have been associated with various psychological effects on the targeted population, ranging from depression and anxiety to post traumatic stress disorder [43,44,45]. Since there are indications that stress phenotypes can alter the SSR, there are several studies putting terrorist attacks under the microscope, since they constitute a major external stressor of the general population. A study, following up on the terrorist attack in New York on 11 September 2001 questioned whether this incident affected the fetal death and SSR in California [46]. The researchers retrieved relevant data and applied interrupted time-series methods to obtain their results. They noticed that the SSR was lower than expected in the cohort born in December 2001 (dropped by 2%) but not in May, June or July 2002, which corresponded to conceptions the following months of the terrorist attacks. Moreover, a significantly higher proportion of male over female fetus losses was noted in October and November of 2001. The authors proposed that the SSR dropped due to male fetal loss caused by the external stressor and not due to decreased male fetus conception, because cohorts conceiving in September, October or November 2001 did not demonstrate altered SSR.

Another study, based also on the terrorist attacks in New York on 11 September 2001, tested the same hypothesis, this time evaluating birth outcomes from New York, USA [24]. The analysis of a total of 717,026 cases revealed that the SSR in January 2002 was significantly lower than the mean SSR of births prior to the attack, while the index eight months after the attack returned to normal levels, implying an increased male fetus loss as potential mechanism. Bruckner et al. also carried out a study assessing birth outcomes, this time across the USA, observing an unexpected increment of male fetus loss odds in September 2001 [47]. They proposed that male fetuses might be more sensitive to maternal corticosteroids produced due to stress after the 20th week of gestation, leading to a higher probability of abortion. A similar study was conducted after the terrorist attacks in Paris in November 2015 [48]. In total, 1,049,057 live births in Paris, France, over a period of 70 months, were assessed (January 2011 to October 2016). Statistical analysis showed an unexpected downward shift of the SSR in December 2015, January 2016 and February 2016.

All the aforementioned studies come to an agreement that terrorist attacks cause a downward shift of the SSR, via inducing stress responses to the pregnant women (Figure 2). This state of stress seems to jeopardize the viability of male embryos, because it was revealed that the probability of male fetus losses was elevated during the following months after the assaults, accompanied by a drop in the SSR. Maternal overproduction and release of corticosteroids, which is a major response to stressors, may disturb the optimal environment male embryos demand, putting their in utero survival at stake.

## 5. The Interplay of Climate Change and SSR

Climate change is a worldwide phenomenon increasingly connected to frequent extreme temperatures in recent years [49]. These temperature fluctuations substantially impact human physiology, resulting in more medical conditions like painful muscle spasms and heat stroke [50]. Despite the importance of this matter, only a limited number of studies have been conducted to explore the effects of temperature changes on SSR.

Initially, a retrospective study examined the impact of annual temperature records on the SSR among three Sami populations: Utsjoki, Inari, and Enontekiö, who are indigenous to Scandinavia [51]. Birth records from parish registries were collected between 1745 and 1890. The study revealed a statistically significant increase of 2.3% in SSR during a warm year, while a significant decrease of 1.4% was observed after a warmer previous year. Due to the contradictory outcomes of concurrent and previous years’ temperatures on SSR, the authors proposed that elevated temperatures could have effects both before conception and during fetal development. Before conception, temperature fluctuations might be linked to reduced motility of Y-sperm, resulting in a lower SSR, or increased testosterone secretion by ovarian follicles, leading to a higher SSR [52,53,54]. Additionally, the authors suggested that higher temperature records might indicate more favourable agricultural conditions for these populations, promoting male births in line with the Trivers-Willard hypothesis [17]. During gestation, it is hypothesized that male fetuses are more susceptible to adverse events, potentially leading to spontaneous loss [55]. However, the present data did not provide evidence to favour any specific mechanism over others.

Similarly, another retrospective study was conducted on Scandinavian populations, including Danes, Finns, Norwegians, and Swedes, from 1878 to 1914 [56]. In this study, annual birth records were analyzed to examine the influence of surface air temperature on SSR. The findings reveal a statistically significant positive association between temperature increase and SSR values. Specifically, for every one-degree Celsius increase in mean temperature, there was an additional male birth for every 1000 female births during that year. Furthermore, the study observed a downward trend in SSR when mean temperatures decreased. This suggests that in utero selection, specifically the selective spontaneous abortions of male fetuses, is the probable mechanism responsible for the negative effect of colder temperatures on SSR [57]. The authors propose several potential mechanisms, such as direct thermal effects, reduced maternal nutrition due to agricultural impacts, and increased exposure to indoor pollutants. However, these mechanisms have yet to be conclusively proven.

Additionally, a 50-year observational study conducted in Finland between 1965 and 2003 observed a significant increase in SSR by 0.06% for every one-degree Celsius increase in ambient temperature beyond normal levels [25]. However, the study authors could not determine whether the temperature effect occurred before or after conception and whether maternal or paternal conditions primarily influenced it. Regarding the preconception period, alterations in sperm characteristics, such as motility and testosterone concentration in ovarian follicles, were suggested as potential pathways [52,54]. During gestation, increased male fetal mortality due to the higher vulnerability of male fetuses to maternal stress levels was once again suggested. To investigate the possible preconceptional effects of air temperature on SSR further, a retrospective study was conducted in the German population from 1946–1995 [58]. Monthly birth records and temperature data for the entire country below an altitude of 800 m were collected. The study found that higher ambient temperatures ten months before birth were statistically associated with an upward shift in SSR. Therefore, the effect observed in this study was preconceptional. An indirect mechanism by which ambient temperatures could influence SSR is increased coital frequency, reducing the time between insemination and ovulation, thus increasing the likelihood of male conceptuses [59]. A study on mice and rats also suggested that an elevation in testicular temperature may lead to alterations in X and Y gametes production during spermatogenesis [60]. Furthermore, parental hormonal levels around conception, particularly elevated levels of testosterone and estrogens, might also contribute to the increase in SSR [61].

More recently, researchers have conducted a study investigating the impact of seasonal variations and ambient temperatures on the SSR within Iceland’s population [62]. The data collected between 1990 and 2014 revealed a statistically significant fluctuation, with the highest SSR observed in June and a decline in January. The study’s authors suggested that the selective elimination of male fetuses may be more prevalent in colder temperatures. Similarly, Fukuda et al. emphasized the potential influence of temperature differences on SSR fluctuations [63]. They discussed the long-term effects of temperature changes on SSR, including an increased sex ratio of fetal deaths and a declining SSR over time. The authors proposed that these changes might affect both the conditions of conception and early pregnancy, resulting in reduced motility of Y-spermatozoa, elevated cortisol levels during the preconception phase, reduced glucose levels during implantation, and increased adrenal androgens at pregnancy [53,61]. The adverse conditions in the uterus could potentially lead to spontaneous abortions of male fetuses, as they are particularly vulnerable to such conditions [64].

In conclusion, climate change and temperature fluctuations, whether extreme or due to seasonal variations, appear to impact the SSR (Figure 2). In fact, in most of the aforementioned cases, higher temperatures increased the SSR, while the selective elimination of male fetuses may be more prevalent in colder temperatures. However, most studies mentioned could not determine the precise effect of temperature on the pre- or post-conception periods, although they did propose various mechanisms to explain the observed trends in SSR.

## 6. Political Affairs and SSR

Political life events, such as elections and referendums, can be a stress factor for the citizens involved [65]. Research by Victor Grech focused on the populations of Cuba and Malta. In Cuba, three migration waves to the USA occurred due to political and economic turbulence, resulting in significant declines in the SSR in the years following these waves [66]. Specifically, three major declines in SSR were observed in 1966, 1980 and 1985, each occurring the year after a massive migrative wave. Similarly, a retrospective analysis in Malta showed a correlation between SSR drops and parliamentary elections [67]. For over 60 years, the country had been divided between two main political parties [68]. Grech analyzed monthly records of SSR in the 13-month periods before and after each election from 1966 to 2013. In the 11 electoral procedures examined, SSR significantly declined in the last seven months before each election. Political polarization acted as a stressor, particularly in the pre-election period. Nevertheless, the study did not specify the exact impact on both parents’ reproductive conditions.

In agreement with the findings above, it was observed that the run of two referendums negatively influenced the SSR in Quebec [69]. On 20 May 1980 and 24 October 1995, the population voted on the potential secession of Quebec from Canada, driven by political and ideological differences with the existing sovereignty [70]. This decision caused significant stress among the population, subsequently impacting the values of SSR. This study recorded annual SSR values for the 5-year periods before and after these events. The findings indicated a negative influence of referendums on SSR. Notably, three months following the second referendum in 1995, a statistically significant decline in SSR was observed, followed by a rapid recovery. The Quebec referendums were considered significant political events that caused intense stress, as the secession from Canada would have had substantial political and economic consequences [71]. Given these circumstances, the author suggested that elevated stress levels could lead to a higher incidence of spontaneous abortions of frail male fetuses, aligning with the Trivers-Willard hypothesis. However, due to the limited information available from the annual record of SSR, further data regarding the specific reproductive factors affected could not be extracted.

To conclude, it has been observed that heightened stress levels during major political events can impact the SSR (Figure 2). Research has shown that male embryos are more vulnerable to maternal stress levels. As a result, during stressful conditions, the frailest male embryos may experience spontaneous abortions. However, the aforementioned studies did not provide additional information regarding pre-conceptional and paternal influences.

## 7. Conclusions and Prospects

The impact of terrorism, climate change, wars, and political affairs on the SSR is a complex and multifaceted issue that warrants further investigation and consideration. Through an in-depth analysis of the available literature, global challenges are seen to influence SSR (Table 1). Specifically, terrorist attacks, low temperatures, and stressful political events have been linked to a decrease in the SSR. Conversely, high temperatures and warfare tend to increase the SSR in numerous scenarios. However, these correlations need to be validated through additional research.

A critical evaluation of the reviewed studies reveals substantial methodological variation that limits comparability and the robustness of conclusions. Sample sizes, study periods, and analytical approaches differ widely. Many investigations rely on retrospective national birth registries, which, although extensive, often fail to adequately account for important confounding variables such as parental age, socioeconomic context, maternal health, and access to reproductive and healthcare services. Variations in temporal baselines and statistical models further contribute to inconsistent findings. These methodological issues highlight the need for more rigorous and standardized study designs that can reliably capture the multifactorial influences on SSR.

Future research should aim to move beyond describing correlations by identifying the specific mechanisms and contextual factors that drive SSR fluctuations. This will require an interdisciplinary framework that integrates demography, environmental health, epidemiology, and the social sciences, as well as biological and behavioral perspectives. Prospective multicenter studies that combine biological markers of stress with socioeconomic and climatic data are essential for clarifying causal pathways. The development of global databases that link reproductive outcomes with indicators of conflict, environmental stress, and political instability could also improve predictive demographic modeling and support early interventions.

By deepening our understanding of how these determinants interact, researchers can better elucidate the complex dynamics that shape SSR. Global challenges have the potential to disrupt the natural balance of male and female births, with long-term implications for population structure and social stability. Addressing these impacts is crucial for promoting gender equality, strengthening public health strategies, and ensuring the well-being of future generations. Continued interdisciplinary collaboration, methodological refinement, and sustained awareness of these interconnections will be vital for managing their broader demographic and societal consequences.

## Figures and Tables

**Table 1:** Summary of major global challenges tending to affect secondary sex ratio (SSR).

Factor	General Effect on SSR	Proposed Mechanisms
Warfare	↑ Increase ^1^	Increased coital frequency or natural selection
Terrorism	↓ Decrease	Increased psychological stress and maternal cortisol levels may reduce male embryo viability
Climate change	↑ Increase (high temperatures)	Secretion of testosterone and estrogens around conception, improved agricultural conditions consistent with the Trivers–Willard hypothesis, and greater coital frequency
	↓ Decrease (low temperatures)	Male spontaneous abortions and reduced motility of Y-bearing sperm
Political affairs	↓ Decrease	Heightened stress during major political events may increase male spontaneous abortions

^1^ Few studies have suggested a negative or any effect of wars on SSR. These associations warrant further validation through comprehensive, large-scale studies aimed at elucidating and mechanistically characterizing the underlying biological pathways.

## Data Availability

Not applicable.
